# Impaired function of endothelial progenitor cells in children with primary systemic vasculitis

**DOI:** 10.1186/s13075-015-0810-3

**Published:** 2015-10-16

**Authors:** Ying Hong, Despina Eleftheriou, Nigel J. Klein, Paul A. Brogan

**Affiliations:** Infection, Immunity, Immunology and Physiological Medicine, Institute of Child Health, University College London, 30 Guilford Street, London, WC1N 1EH UK

**Keywords:** Endothelial progenitor cells, Vasculogenesis, Matrigel, Inflammation, Vasculitis

## Abstract

**Introduction:**

Previously, we demonstrated that children with active systemic vasculitis (SV) have higher circulating CD34 + CD133 + KDR+ endothelial progenitor cells (EPC); the function of these EPCs, and their relationship with disease activity in vasculitis remains largely unexplored. We hypothesized that although EPC numbers are higher, EPC function is impaired in active SV of the young. The aims of this study were therefore to: 1. investigate the relationship between disease activity and EPC function in children with SV; and 2. study the influence of systemic inflammation on EPC function by investigating the effects of hyperthermia and TNF-α on EPC function.

**Methods:**

We performed a cross-sectional study of unselected children with SV with different levels of disease activity attending a single center (Great Ormond Street Hospital, London) between October 2008 and December 2014. EPCs were isolated from peripheral blood of children with SV, and healthy child controls. EPC function was assessed by their potential to form colonies (EPC-CFU), and ability to form clusters and incorporate into human umbilical vein endothelial cell (HUVEC) vascular structures in matrigel. The effects of hyperthermia and TNF-α on EPC function were also studied.

**Results:**

Twenty children, median age 12-years (5–16.5; nine males) were studied. EPC-CFU and the number of EPC clusters formed on matrigel were significantly reduced in children with active vasculitis compared with healthy controls (p = 0.02 for EPC-CFU; p = 0.01 for EPC cluster formation). Those with active vasculitis had lower EPC-CFU and EPC cluster formation than those with inactive disease, although non-significantly so. In addition, EPC incorporation into matrigel HUVEC networks was lower in children with SV compared with healthy children, irrespective of disease activity. Ex-vivo pre-treatment of EPC with hyperthermia impaired EPC function; TNF-α down-regulated EPC expression of CD18/CD11b and resulted in decreased incorporation into HUVEC networks.

**Conclusions:**

Whilst our previous work showed that circulating CD34 + EPC numbers are well preserved, this study revealed that EPC function is significantly impaired in children with vasculitis. It is possible that the chronic inflammatory milieu associated with vasculitis may impair EPC function, and thus contribute to an unfavourable balance between endothelial injury and repair. The mechanism of this remains to be established, however.

**Electronic supplementary material:**

The online version of this article (doi:10.1186/s13075-015-0810-3) contains supplementary material, which is available to authorized users.

## Introduction

Systemic vasculitis (SV) of the young is characterised by systemic inflammation, endothelial activation, and (in some cases) necrosis of blood vessel walls leading to multi-organ injury [1, 2]. Endothelial activation and injury are central to the pathogenesis of SV with increased endothelial cell adhesion molecule expression, and a switch to a prothrombotic endothelial phenotype, both of which contribute to the vascular pathology of SV [3, 4]. In addition to this severe endothelial injury, it has been suggested that endothelial repair processes may also be impaired [5, 6], and thus SV could be particularly damaging to the cardiovascular system due to an unfavourable imbalance between endothelial injury and repair, as is the case in other diseases targeting the endothelium [7, 8].

It is now known that recruitment of bone marrow-derived endothelial progenitor cells (EPC) represents an important mechanism of endothelial repair [9]. These EPCs may play an important role in endothelial maintenance and vascular healing in health and disease, and are emerging as important biomarkers of prognosis in atherosclerosis [10, 11]. Previous studies have employed two main methods to study EPCs in humans: 1) flow cytometric quantification of circulating CD34+ haematopoietic progenitors, usually co-expressing additional surface markers including CD133 and/or vascular endothelial growth factor receptor 2 (VEGFR2) [12]; and 2) quantification of circulating myeloid-derived EPC colony forming units (EPC-CFU) in angiogenic medium [12]. Little is known, however, about the impact of SV on these repair processes, despite concerns that vascular dysfunction is an important late sequelae of vasculitis in children [13, 14] and adults [15].

We have previously postulated that there is an unfavourable balance between endothelial injury and repair in children with SV [16], and that aging and dyslipidaemia also influence this balance [17]. We previously demonstrated evidence of excessive endothelial injury in children with SV: elevated numbers of circulating endothelial cells (CECs) [16], and increased circulating endothelial microparticles (EMPs) [16, 18]. It is possible that children mount an attempted adaptive response to this excessive endothelial injury, since we also observed increased circulating CD34 + CD133 + VEGFR2 + EPCs using flow cytometric analysis of peripheral blood monocytes in children with active vasculitis [16]. The functional relevance of this possible adaptive haematopoietic EPC response in paediatric SV has not yet been established, however. In the light of our previous findings, we hypothesised that the significant inflammatory milieu associated with SV would adversely affect EPC function, resulting in defective colony formation, adhesion and ability to integrate into vascular networks. The aims of the current study, therefore, were to: 1) investigate the relationship between disease activity and EPC function in children with SV; and 2) to study the potential influence of systemic inflammation on EPC function by investigating the effects of hyperthermia and TNF-α on EPC function *in vitro*.

## Methods

### Patients and controls

We performed a cross-sectional study of unselected children with SV with different levels of disease activity attending a single center (Great Ormond Street Hospital NHS Foundation Trust, London) between October 2008 and December 2014. Parental consent was obtained for all children involved in the study, which was approved by the Institutional Ethics Committee (see acknowledgements for details). Inclusion criteria for children with SV were as follows: age <18 years, a diagnosis of SV confirmed by histopathologic and/or arteriographic assessment, and secondary causes of vasculitis excluded (infection, other connective tissue disease, or malignancy). Control samples were obtained from healthy sex- and age-similar children; adult healthy controls were members of staff within our laboratory (median age 33 years, range 22–35 years; one female). All participating subjects provided written, fully-informed consent.

Vasculitis subtype was classified using the EULAR/PRINTO/PReS classification criteria for paediatric vasculitides [19] for: polyarteritis nodosa (PAN), granulomatosis with polyangiitis (GPA, formerly Wegener’s granulomatosis), and Takayasu arteritis (TA). Kawasaki disease (KD) was defined as patients fulfilling at least five of six of the American Heart Association criteria for KD [20]. Microscopic polyangiitis (MPA) was defined according to the Chapel Hill definitions [21]. Patients with Behçet’s disease (BD) fulfilled the International Study Group for Behçet’s Disease criteria [22]. Disease activity was assessed using the Paediatric Vasculitis Activity Score (PVAS) [16, 19, 23, 24] which is shown in detail in Additional file 1 (Paediatric Vasculitis Activity Score) and Additional file 2 (glossary and scoring for PVAS). Active vasculitis was defined as a score greater than zero for PVAS items attributable to vasculitis that newly appeared or worsened during the preceding four weeks, and for which other causes such as infection were excluded [16, 24]. Patients in remission had a PVAS of 0/63. Routine laboratory markers [erythrocyte sedimentation rate (ESR) normal range 0–10 mm/h, and C-reactive protein (CRP) normal range < 20 mg/L] levels provided adjunctive information regarding systemic inflammation related to systemic vasculitis activity.

### Immunomagnetic bead extraction of circulating endothelial cells from peripheral blood

CECs were extracted from whole blood using CD146-coated immunomagnetic beads using an international consensus protocol [25, 26]. CECs extracted in this way were counted using a Nageotte chamber on a fluorescence microscope and were defined as *Ulex europaeus* lectin (Sigma-Aldrich, Doset, UK) bright cells that were > 10 μm in size, with five or more magnetic beads attached.

### Functional assay of endothelial progenitor cells

#### Endothelial progenitor cell colony-forming units

**Fig. 1:**
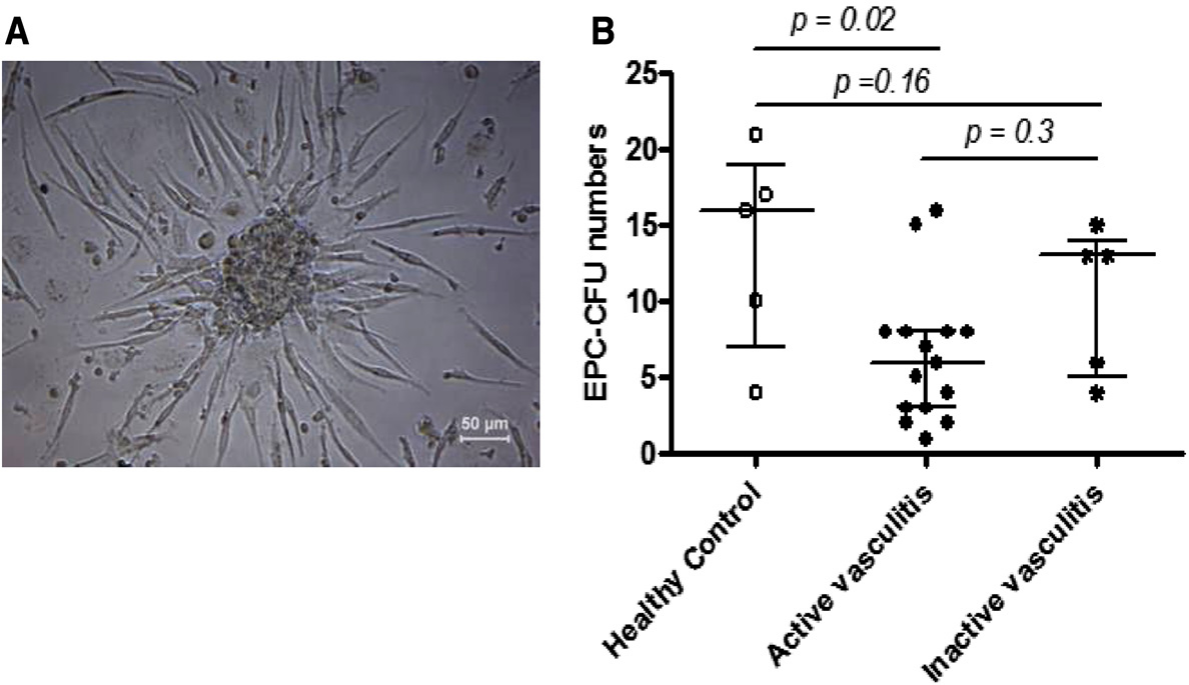
Reduced endothelial progenitor cell-colony forming units (EPC-CFU) in children with active systematic vasculitis. **a** Representative image of EPC-colony forming unit (EPC-CFU from a healthy control child). **b** Comparison of EPC-CFU in children with active and inactive systematic vasculitis and child healthy controls. EPC-colony forming EPC-CFUs were reduced in 15 children with active disease compared to healthy control children but not in five children with inactive disease compared to healthy child controls (*p* = 0.3). The Kruskal-Wallis test was used to examine overall differences between the study groups followed by the Mann–Whitney *U* test. P values < 0.05 were considered significant

EPC-CFUs were cultured with slight modification from methods previously described [9, 27]. Briefly, peripheral blood mononuclear cells (PBMC) were isolated by density centrifugation (Lymphoprep TM, Axis Shield, Dundee, UK). After purification with three washing steps, 2 × 10^6^ PBMCs were plated on fibronectin-coated 24-well plates. Cells were cultured and maintained in endothelial growth media (EGM-2) culture medium supplemented with growth factors according to the manufacturer’s recommendations (PromoCell, Heidelberg, Germany), plus 20 % foetal calf serum (FCS) and 40 ng/ml of vascular endothelial growth factor (VEGF). After four days of culture, non-adherent cells were removed by washing with phosphate buffered saline (PBS). In some experiments TNF-α (10–50 ng/ml) was added to the media. Culture medium was changed to maintain the cells in culture until day 7. The numbers of myeloid-EPC colony forming units (EPC-CFU), characterized by a cluster of cells surrounded by elongated spindled-shaped cells, were counted manually in a minimum of two wells in 24-well plates by two independent observers who were unaware of the clinical profiles of the patients from whom the cells were derived. Results were expressed as average number of EPC-CFUs per well. Fig. 1a shows a representative EPC-CFU on day 7 from EPCs isolated from a five-year-old healthy control child. In selected samples, uptake of Di-I-acetylated low-density lipoprotein (Di-IAcLDL, Invitrogen, Paisley, UK) was confirmed, in line with other studies of this nature [10, 27].

#### Matrigel assays: EPC cluster formation and incorporation assays

Matrigel assays were employed to examine the ability of EPC to incorporate into endothelial capillary networks (hereafter referred to as the EPC-incorporation assay) [28] and the ability of EPC to form clusters (clusters of more than ten cells), a measure of EPC sprout formation (hereafter referred to as the EPC-cluster formation assay) [29]. Human umbilical vein endothelial cells (HUVEC; PromoCell) were cultured in EGM-2 medium supplemented with 2 % FCS, hydrocortisone, fibroblast growth factor (FGF-2), VEGF, R^3^-insulin-like growth factor-1, epidermal growth factor, heparin, ascorbic acid, gentamycin and amphotericin B, as supplied by the manufacturer (PromoCell) at 37 °C in 5 % CO_2_ in a humidified incubator. HUVECs at 80 % confluency (passages 2–4) were used for experiments. Growth factor reduced Matrigel Matrix (Becton Dickinson Labware, Oxford, UK) was thawed and placed in 96-well plates at 37 °C for 30 min to solidify. EPCs were labeled with 2 μg/ml of Di-I-LDL to distinguish them from HUVECs.

For the EPC-cluster formation assay, 10,000 EPCs were plated in Matrigel; for the EPC-incorporation assay, DiI-labeled EPCs (3,000/well) were co-plated with HUVEC (10,000/well) on top of a solidified Matrigel layer and cultured at 37 °C for 18–20 h with EGM-2 medium.

Incorporation of EPC into capillary networks of HUVEC on Matrigel or EPC cluster formation was then examined with fluorescence microscopy. Five independent fields were assessed for each well and the mean numbers of EPCs incorporated into the HUVEC capillary networks and EPC clusters/x 100 fields were determined.

### Effects of TNF-α and hyperthermia on EPC adhesion and incorporation into HUVEC networks

#### EPC adhesion assay

EPC adhesion to fibronectin and HUVECs was assessed. EPCs were labeled with Di-I-LDL and 1 × 10^5^ cells were placed on fibronectin-coated 96-well plates or on a monolayer of HUVECs pretreated with TNF-α (50 ng/ml) for six h, and incubated for one h at 37 °C. Non-attached cells were gently removed by washing with PBS, and adherent EPCs were fixed with 4 % paraformaldehyde and counted in ten random fields.

#### XXT assay of cell viability in response to hyperthermia

Cell viability under different experimental conditions was determined by XTT (xylin tetrazolium) assay, which is based on the ability of metabolically active cells to reduce the tetrazolium salt XTT to orange coloured compounds of formazan. EPC or HUVEC (1 × 10^4^) were plated into 96-well plates in EGM-2. After 24 h at 37 °C, cells were incubated at 39 °C for 2, 4, 18 h, then replaced in a 37 °C incubator for up to 18 h of total culture time. XTT was added to the culture medium and incubated at 37 °C for four h. The formazan product was read at a wavelength of 545 nm with reference wavelength at 690 nm.

### Statistical analysis

All *in vitro* experiments were performed in triplicate unless otherwise stated, and values are presented as mean ± standard error of the mean unless otherwise specified. Statistical differences for in vitro experiments between groups were determined by two-way analysis of variance (ANOVA), followed by unpaired two-tailed *T*-test. Patient demographics are summarized as median and range. The Kruskal-Wallis test was used to examine overall differences between the study groups followed by the Mann–Whitney *U* test. P-values of less than 0.05 were regarded as significant. All analysis was performed using SPSS version 22.

## Results

### Patients and controls

We studied 20 unselected consecutive patients with a diagnosis of SV; the median age at time of study was 12.0 (range 9.0–16.5) years; six patients were male. There were five paediatric healthy controls (two male, three female) of median age four years (range, 3.6-10 years). Fifteen children had active SV (n = 8 PAN; n = 4 GPA; n = 1 EGPA; n = 1 KD; n = 1 unclassified but biopsy proven SV), with median PVAS of 4/63 (range 4–26/63); five children had inactive SV (n = 4 with GPA; and n = 1 Behçet’s disease) with a PVAS of 0/63 in all. The clinical features of the vasculitis patients are summarized in Table 1. Median follow up for those with active disease was nine months (6–36 months); median follow up for those with inactive disease was 12 months (6–24 months).

**Table 1:** Patient characteristics, baseline laboratory parameters, circulating endothelial cell counts and treatment in 20 children with systemic vasculitis

Demographic data	Active PSV (*n* = 15)	Inactive PSV (*n* = 5)
Median age, years (range)	12 (7–16.5)	12 (9–11.6)
Sex M/F	5 M:10 F	1 M:4 F
Classification	n = 8 PAN; n = 4 GPA; n = 1 EGPA; n = 1 KD; n = 1 unclassified SV	n = 4 GPA; n = 1 Behçet’s disease
ESR, mm/h (median, range)	73 (11–137)	3 (3–37)
CRP, mg/L (median, range)	18.5 (5–270)	5 (5–16)
CEC count, cells/ml (median, range)	88 (8–420)	16 (0–40)
Treatment (n)		
Cyclophosphamide	5	2
Corticosteroids	11	3
MMF	1	2
Azathioprine	0	1
Colchicine	0	1
Rituximab	1	0

Classification of the vasculitic syndromes was based on the recent EULAR/PRINTO/PRES classification criteria for pediatric vasculitis [19]. KD was identified based on five of six of the American Heart Criteria. Immunomagnetic bead extraction was used for enumeration of circulating endothelial cells (CEC). Treatments summarised were received at any point of the patients’ disease course. *PAN* polyarteritis nodosa, *KD* Kawasaki disease, *GPA* granulomatosis with polyangiitis, *EGPA* eosinophilic granulomatosis with polyangiitis, *ESR* erythrocyte sedimentation rate, *CRP* C-reactive protein, *MMF* mycophenolate mofetil

Patients with clinically active vasculitis had evidence of endothelial injury, with an increased median CEC count of 88 (8–420) cells/ml, and systemic inflammation as indicated by a median ESR of 73 (11–137) mm/h and a CRP of 18.5 (5–270) mg/L; for children with inactive disease median CEC count was 16 (0–40) cells/ml, median ESR was 3 (3–37) mm/h, and median CRP was 5 (5–16) mg/L. For healthy controls there was a median CEC count of 24 (0–80) cells/ml; these healthy paediatric controls did not have CRP or ESR performed.

### EPCs from children with active vasculitis have decreased colony-forming capacity

EPC-CFUs were reduced in 15 children with active vasculitis (median 6/well; range 1-16/well) compared to healthy control children (median 16/well; range 4-21/well), p = 0.02 and also (non-significantly) lower than children with inactive vasculitis (median 13/well; range 4-15/well), p = 0.16 (Fig. 1b). There was no significant difference in EPC-CFU among the five children with inactive vasculitis compared to healthy child controls (p = 0.3; Fig. 1b).

### EPCs from children with active vasculitis demonstrate decreased cluster formation on Matrigel

**Fig. 2:**
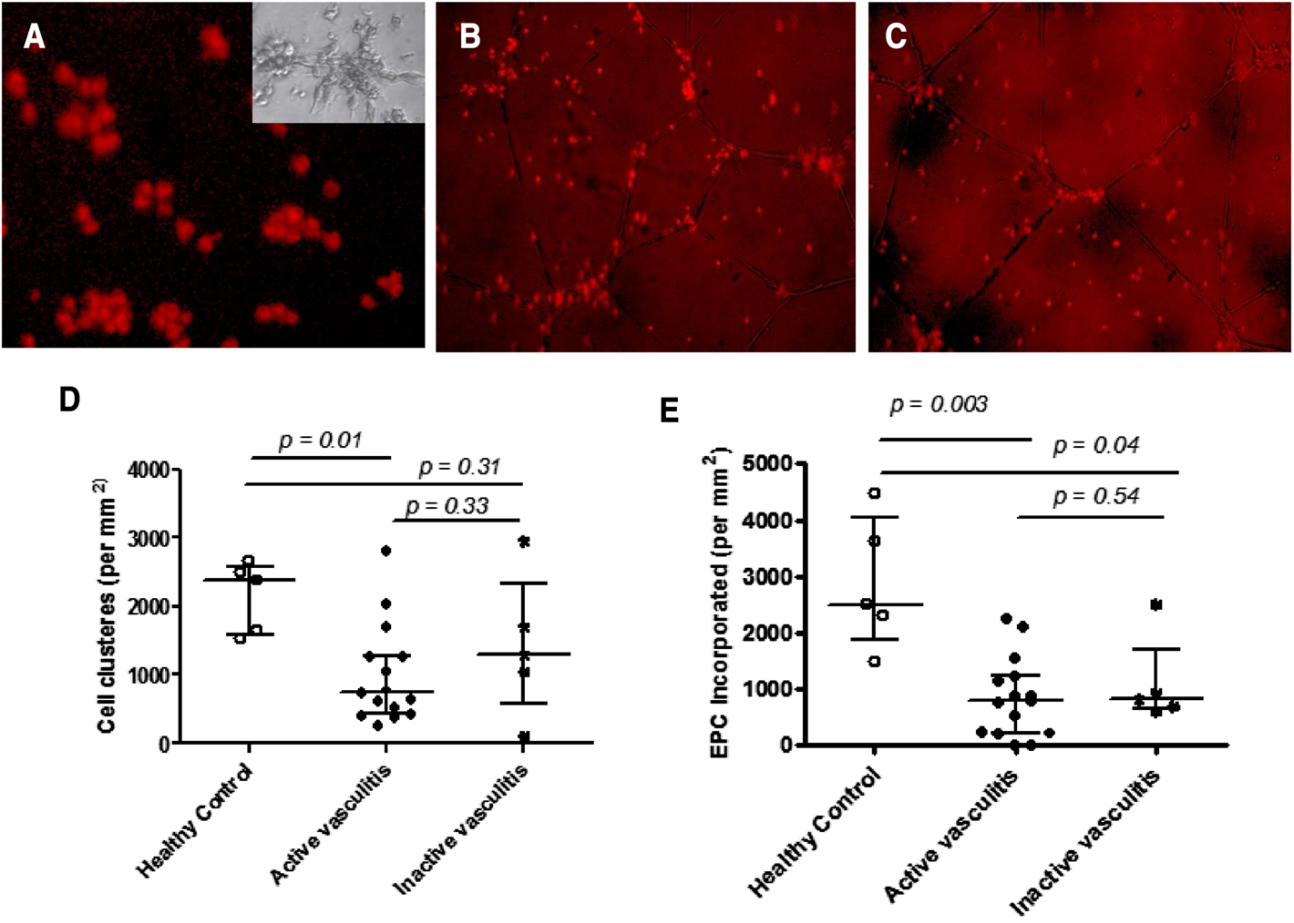
Reduced incorporation of endothelial progenitor cells (EPCs) into endothelial capillary network and cluster formation on Matrigel in children with systemic vasculitis. **a** Representative image of EPC cluster formation on Matrigel. The *inset* shows an example of a sprouting EPC. **b** and **c** Representative images of the incorporation of EPCs from a healthy child (**b**), and a child with active granulomatosis with polyangiitis (**c**) into an endothelial capillary network on Matrigel. **d** Comparison of the number of EPC clusters with active and inactive systemic vasculitis and child healthy controls. The number of EPC clusters was reduced for 15 children with active disease compared with healthy control children (*p* = 0.01); cells clusters in the inactive vasculitis group did not differ significantly from healthy controls. **e** EPC incorporation into HUVEC capillary networks on Matrigel in children with active and inactive systematic vasculitis and child healthy controls. Compared to five healthy control children, the number of EPC incorporated into HUVEC vascular networks on Matrigel was decreased for 15 children with active vasculitis (*p* = 0.003) and five children with inactive vasculitis (*p* = 0.04). The Kruskal-Wallis test was used to examine overall differences between the study groups followed by the Mann–Whitney *U* test. P values <0.05 were considered significant. *HUVEC* human umbilical vein endothelial cell

EPCs cultured for seven days were plated on Matrigel-coated wells to assess EPC cluster formation (clusters of EPCs containing more than ten cells), an inherent angiogenic functional property of EPCs and a prelude to vascular sprouting (Fig. 2a, and insert). EPC cluster formation was significantly lower in 15 children with active vasculitis (median 738/mm^2^; range 0–2,813) compared to five healthy control children (median 2,386 /mm^2^; range 1,522–2,652); p = 0.01 and also lower (non-significantly) than children with inactive vasculitis (median 1,295 /mm^2^; range 100–2,955; p = 0.31; Fig. 2b). There was no significant difference in EPC cluster formation between children with inactive vasculitis and healthy controls (p = 0.33).

### EPCs from children with active vasculitis demonstrate decreased incorporation into HUVEC networks

We next performed a Matrigel assay to investigate the ability of EPCs to incorporate into HUVEC vascular networks. Co-culture of EPCs (from systemic vasculitis patients or healthy controls) and HUVECs on Matrigel led to the formation of vascular tubule networks (Fig. 2c and d). However, as summarised in Fig. 2e, the number of EPC incorporated into HUVEC vascular networks on Matrigel was decreased for 15 children with active vasculitis (median 795/mm^2^; range 0-2,235/mm^2^; p = 0.003) and five children with inactive vasculitis (median 814/mm^2^; 596-2,500/ mm^2^; p = 0.04), compared to five healthy control children (median 2,500/mm^2^; range 1,478-4,478/mm^2^).

### TNF-α reduces EPC incorporation into HUVEC networks

**Fig. 3:**
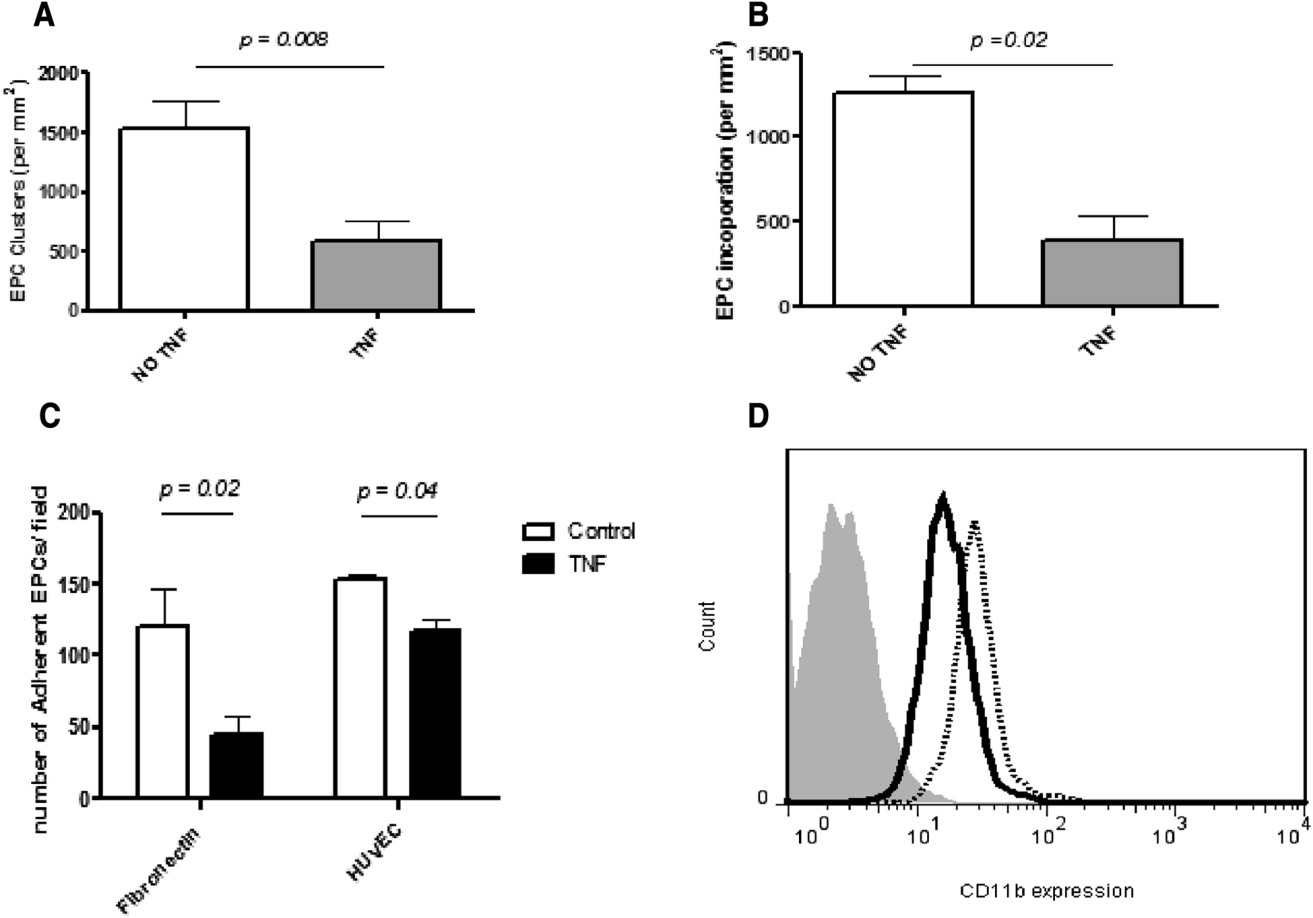
The effect of tumour necrosis factor (TNF-α) on EPC vasculogenesis, adhesion and CD11b expression. EPCs were pre-treated with 10 ng/ml TNF-α for three days before plating for the Matrigel assay to study the chronic effect of TNF-α on EPC function. **a** and **b** Comparison of EPC with/without pre-treatment of TNF-α on cluster formation (**a**) and incorporation into HUVEC capillary networks (**b**) on Matrigel. The numbers of EPC clusters (**a**) were lower after pre-treatment with TNF-α (*p* = 0.008). EPC incorporation into HUVEC vascular networks on Matrigel (**b**) was also significantly reduced after pre-treatment with TNF-α (*p* = 0.02). **c** Effect of TNF-α on EPC adhesion to fibronectin or monolayers of activated HUVECs. EPCs were labeled with Dil-I-LDL and allowed to adhere for one h to fibronectin or a monolayer of TNF-α-activated HUVECs. Pretreatment with TNF-α resulted in significantly reduced EPC adherence to fibronectin (*p* = 0.02) or activated HUVEC monolayers (pretreated with TNF-α 50 ng/ml for six h; *p* = 0.04). **d** Representative flow cytometric profile of CD11b expression by EPC after pretreatment with TNF-α 10 ng/ml for three days (thick black tracing) compared with control (no TNF-α pre-treatment; broken tracing). *EPC* endothelial progenitor cells, *HUVEC* human umbilical vein endothelial cell

We next assessed the effect of TNF-α on the function of EPCs, since this inflammatory cytokine has been implicated in the pathogenesis of many forms of SV in children and adults [30, 31]. EPC were pre-treated with 10 ng/ml TNF-α for three days of EPC culture, then EPC were collected and the Matrigel assay was performed. The numbers of EPC incorporated into HUVEC vascular networks, and EPC clusters on Matrigel were significantly reduced after pre-treatment with TNF-α (p = 0.02 and p = 0.008, respectively, Fig. 3a and b). In contrast, TNF-α (10–100 ng/ml) increased tube formation of HUVECs, similar to the effect of VEGF, an effect that was blocked by 10 μg/ml infliximab (a chimeric monoclonal antibody against TNF-α; data not shown).

### TNF-α reduces EPC adhesion by decreasing CD18/CD11b expression

We then examined whether the inhibitory effect of TNF-α on EPC vasculogenesis was due to alteration of the adhesiveness of cultured human EPCs to the activated endothelial cells and/or the extracellular matrix. EPCs were again pre-treated with 10 ng/ml TNF-α for three days, detached with 0.25 % trypsin, and then re-plated onto fibronectin-coated dishes or HUVEC monolayers. EPCs pre-treated with TNF-α exhibited a significant decrease in the number of adhesive cells at 60 min compared with control (Fig. 3c): adhesion to fibronectin at baseline 120 ± 50 versus 44.7 ± 17.9 post exposure to TNF-α; p = 0.02 and adhesion to activated HUVEC monolayers at baseline 152.7 ± 10.5, versus 118.7 ± 17.4 after TNF-α, p = 0.04.

Since EPCs are known to migrate to, and accumulate at, the site of tissue injury where they express complementary sets of surface receptors (CD11b/CD18, CD54) that could influence adhesion at these sites [32], using flow cytometry we next investigated whether TNF-α would alter the expression of these surface receptors on EPC. Pre-treatment with TNF-α down regulated the expression of CD11b (Fig. 3d), but increased that of CD54, and had no effect on CD146 or CD34 (data not shown).

### Prolonged hyperthermia impairs HUVEC angiogenesis and reduces EPC cluster formation

**Fig. 4:**
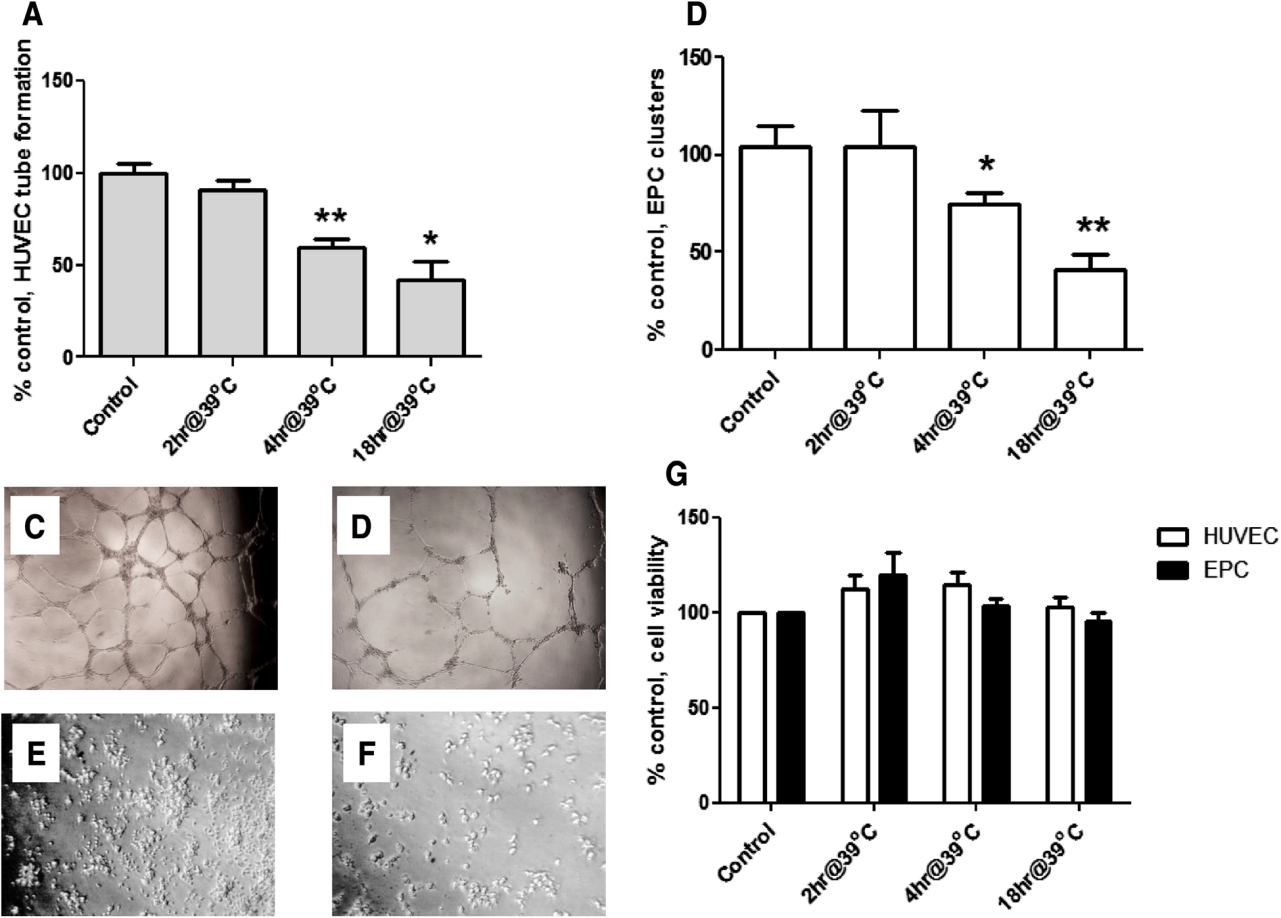
Effect of hyperthermia on endothelial progenitor cell (EPC) vasculogenesis, human umbilical vein endothelial cell (HUVEC) tube formation, and viability. After EPC or HUVECs were plated on Matrigel for 1 h, cells were cultured at 39 °C for 2–18 h; after 2, 4 and 18 h, EPC clusters and HUVEC tube branches were counted in 5/x100 fields, and the percentage of EPC clusters and tube formation (compared to control) was plotted for each time point. **a** Hyperthermia inhibited HUVEC tube formation and **b** EPC cluster formation on Matrigel. **c**-**f** Representative images of HUVEC capillary networks and EPC cluster formation on Matrigel at different temperatures. c HUVEC network at 37 °C. Exposure to 39 °C for 4 h inhibited HUVEC capillary network formation (**d**). **e** EPC cluster formation on Matrigel at 37 °C was also reduced by incubation at 39 °C for 4 h (**f**). **g** Effect of hyperthermia on cell viability. Cell viability was measured by XTT assay 2-18 h after heating. Cell viability of untreated cells is assumed as 100 %. Incubation at 39 °C at 2, 4 and 18 h did not cause cell death. All data represent the mean ± SD of three experiments. Statistical differences between heat treatment and control were determined by two-way analysis of variance (ANOVA), followed by paired two-tailed *T*-test. ∗ = *P* < 0.05; ** = *P* < 0.01. *XTT* xylin tetrazolium

Sustained/prolonged pyrexia is an important clinical feature of virtually all chronic inflammatory diseases, including SV. We therefore investigated the influence of hyperthermia on HUVEC vascular network formation and EPC cluster formation. HUVECs were initially incubated in Matrigel for 60 min at 37 °C to allow their adhesion, followed by exposure to hyperthermia (39 °C) for 2–18 h. As shown in Fig. 4a, prolonged exposure to hyperthermia resulted in a significant decrease in HUVEC tube formation at 4 and 18 h. DiI-labeled EPCs plated on Matrigel at 39 °C for 2–18 h demonstrated decreased cluster formation by 4 h (Fig. 4b). To investigate whether these effects were the result of hyperthermia-induced cytotoxicity, we evaluated HUVEC and EPC viability after exposure to hyperthermia using a colorimetric assay; EPC or HUVECs incubated under heat at different time points did not exhibit signs of increased cell death (Fig. 4c).

## Discussion

In this study we demonstrated impaired endothelial repair capacity of EPC in children with SV. Using *in vitro* assays recapitulating *in vivo* morphogenesis events we have shown that EPC adherence, colony formation, and integration into endothelial networks were significantly impaired in children with active SV compared to patients with inactive vasculitis and healthy child controls. Our findings could suggest an impairment of endothelial repair capacity. As in adults with SV and other vascular disorders, it would be important to understand if this impaired endothelial repair response could contribute to cardiovascular sequelae.

Despite the lack of a consensus definition for an EPC [33], alterations in EPC biology have been linked to several human illnesses associated with cardiovascular morbidity and risk [34, 35]. Very little is currently known about EPC biology in patients with SV, although studies suggest that chronic inflammatory autoimmune diseases including rheumatoid or psoriatic arthritis [36], systemic lupus erythematosus [6, 37], and systemic sclerosis [38] (amongst others) are associated with impairment in EPC function that could contribute to the excessive cardiovascular morbidity associated with these diseases. In SV, non-specific effects of chronic inflammation including pro-inflammatory cytokine milieu [39] and sustained pyrexia [40] could influence EPC biology in an adverse way, particularly when associated with other factors associated with the burden of these diseases such as uraemia [41], hypertension [42], dyslipidaemia [43], and impaired glucose tolerance [44]. Since a hallmark of SV is inflammation targeting endothelial cells, irrespective of the cause, impairment of EPCs may be of particular importance clinically since this would contribute further to an unfavourable balance between endothelial injury and repair.

Studies of EPCs in adults with SV relate mainly to anti-neutrophil cytoplasmic autoantibody (ANCA) associated vasculitis (AAV), and so far have provided apparently conflicting results, probably the result of patient and methodological heterogeneity and/or differences in treatment. Holmen et al. demonstrated that the numbers of EPC-CFU were significantly decreased during active GPA as compared with remission, and suggested that impairment of EPC function was mediated in part by what they referred to as inflammatory circulating endothelial cells [45]. In contrast, De Groot et al. demonstrated no significant difference in circulating CD34+ haematopoietic stem cells and EPC-CFU numbers in patients with active AAV compared with healthy control levels [46]. Both these EPC parameters, however, increased after the institution of immunosuppressive therapy and disease remission in GPA patients. Závada et al. confirmed the observation of Holmen et al. of low EPC-CFU in adults with AAV, particularly in uraemic patients; but in contrast to the study of de Groot et al. found that neither institution of treatment nor entering remission increased the number of EPC-CFU [47]. In a separate study the same group went on to demonstrate that patients with AAV who had low EPC-CFU at first presentation had significantly higher rates of early disease relapse, for the first time suggesting that EPC levels might be important prognostically in AAV [48].

Only two studies have examined changes in EPCs in children with vasculitis [16, 49]. In contrast to adults with AAV, we have previously shown that children with active SV respond to endothelial injury by release of CD34 + CD133 + KDR+ EPCs into the circulation, possibly as an adaptive response to endothelial injury caused by vasculitis [16]. This observation was consistent with the findings of Nakatani et al. who observed a rise in circulating CD34 + CD133+ EPCs in the sub-acute phase of KD complicated by coronary artery lesions [49]. The findings of our current study, however, emphasise that CD34+ positive haematopoietic EPCs represent a different cell population from the monocytic EPCs that undoubtedly represent the cells that form the colonies and Matrigel clusters from PBMCs as defined in our present study, and many other studies [33]. The present findings, taken together with our previous studies [16, 18] suggest that in children with active vasculitis there is an unfavourable balance between endothelial injury and repair since there is increased endothelial injury reflected by an increase in CECs and EMPs and an additional disturbance in cells efficient for repair.

Our data suggest that chronic inflammation per se could account for impaired monocytic EPC function since we established that both TNF-α and hyperthermia impaired EPC function, in agreement with previous studies demonstrating an adverse effect of TNF-α on EPC function in adults with RA [50]. Indeed, there is also a considerable amount of indirect evidence supporting a role of TNF-α in the pathogenesis of systematic vasculitis [51–53]. As well as confirming this adverse effect of TNF-α on EPCs, for the first time we also demonstrated that TNF-α downregulated the expression of CD11b on EPC, providing a mechanism for reduced adhesion of EPC into vascular networks. This observation would not preclude alternative mechanisms, such as accelerated EPC senescence, that could also contribute to this EPC dysfunction [54–57].

Our findings have several implications. We now have the unique ability to explore the endothelium non-invasively, defining an imbalance between endothelial damage and repair capacity for children with SV. This multi-marker approach combining CEC, EMP and EPC measurement provides novel insights into endothelial dysfunction and moving forward could allow us to explore the prognostic relevance of this approach in individual patients studied longitudinally in multicentre collaborative studies. Future therapeutic strategies aiming to improve endothelial function, employing for instance statins, ACE inhibitors or PPAR-gamma agonists (amongst other potentially important drugs), could target the selective reduction of endothelial injury and/or the promotion of regenerative mechanisms, but ideally would address both sides of the injury/repair equation. At the individual level, such personalised therapeutic options could help reduce late cardiovascular morbidity associated with childhood vasculitis [13]. In that context it would be of considerable interest to prospectively examine the influence of altered endothelial repair responses in children with SV on late structural arterial injury [13], an ongoing concern for survivors of other childhood vasculitides such as KD [58].

Our study has limitations. Systemic vasculitis is rare in children and consequently the study population was relatively small and heterogeneous; that said, we did not observe any obvious difference in EPC function between different forms of vasculitis in this study, and have previously demonstrated that downstream endothelial injury processes are similar across different forms of paediatric vasculitides [16, 18, 25, 59]. Additionally, the longitudinal effect of treatment on EPC responses was not examined in individual patients, but clearly it would be advantageous to do so in the future. Other limitations relate to the poor standardization of assays for EPCs, an inherent problem for all studies of this nature. Methodological issues and confusion created by the various definitions currently in use remain a major cause of apparent conflicting results regarding changes in EPC biology in human diseases. Standardisation of the methodology used to quantitate EPCs including an exact and consensus definition for EPCs is urgently needed to facilitate future studies [50].

## Conclusions

In summary, two key findings from our study and previous work in this area are: 1) circulating EPCs are increased in response to active vasculitis in the young, a finding that contrasts with adults with SV; and 2) EPC function (*ex vivo*) is impaired. Whilst the clinical relevance of this observation remains undetermined, our findings could indicate an impaired endothelial repair capacity that, in the presence of ongoing endothelial injury, may further contribute to endothelial dysfunction. Future studies are now required to address the clinical implications of these observations in terms of late cardiovascular morbidity and the influence of treatment on EPC biology in children with systemic vasculitis.

## Additional files

Additional file 1:
**Paediatric Vasculitis Activity Score.** (PDF 51 kb) Additional file 2:
**Glossary and scoring for PVAS.** (PDF 61 kb)

## Abbreviations

EPC: Endothelial progenitor cells
EPC-CFU: EPC colony forming unit
HUVEC: Human umbilical vein endothelial cell
SV: Systemic vasculitis
