# Supplementary material for: Impaired function of endothelial progenitor cells in children with primary systemic vasculitis
Source: Arthritis Res Ther. 2015 Oct 16;17:292. doi: 10.1186/s13075-015-0810-3 (PMC4609146; doi:10.1186/s13075-015-0810-3)
Supplement: Additional file 1: — Paediatric Vasculitis Activity Score. (PDF 51 kb) [file 13075_2015_810_MOESM1_ESM.pdf]

PAEDIATRIC VASCULITIS ACTIVITY SCORE

○ Tick "**Active**" box **only** if abnormality due to active vasculitis is newly present or worse over the last 4 weeks or persists for less than 3 months. After that, if ALL items are persistent and represent smouldering/low grade/grumbling disease, and there are no new/worse features, please tick the box at the bottom right corner. At the very first assessment all active items are considered as active/worse. If there are no abnormalities in a system, please tick the "**None**" box. For items present longer than 3 months refer to the Vasculitis Damage Index to score damage.

	None	Active
**1. General**	○	
Myalgia		○
Arthralgia or arthritis		○
Fever ≥ 38.0 ^{0}C		○
Weight Loss ≥ 5% body weight		○
**2. Cutaneous**	○	
Polymorphous exanthema		○
Livedo		○
Panniculitis		○
Purpura		○
Skin nodules		○
Infarct (nail edge lesion, splinter haemorrhage)		○
Ulcer (full-thickness necrosis)		○
Gangrene (extensive necrosis)		○
Other skin vasculitis (specify below)		○
**3. Mucous membranes/eyes**	○	
Mouth ulcers/granulomata		○
Genital ulcers		○
Adnexal inflammation		○
Significant proptosis		○
Red eye (Epi)scleritis		○
Red eye conjunctivitis/ blepharitis/keratitis		○
Uveitis		○
Blurred vision		○
Sudden visual loss		○
Retinal vasculitis/retinal vessel thrombosis/ retinal exudates/haemorrhages		○
**4. ENT**	○	
Nasal discharge/crusts/ulcers/granuloma		○
Paranasal sinus involvement		○
Subglottic stenosis/ hoarseness /stridor		○
Conductive hearing loss		○
Sensorineural hearing loss		○
**5. Chest**	○	
Wheeze or expiratory dyspnea		○
Endobronchial/endotracheal involvement		○
Nodules or cavities		○
Pleural effusion/pleurisy		○
Infiltrate		○
Massive haemoptysis/Alveolar haemorrhage		○
Respiratory failure		○

	None	Active
**6. Cardiovascular**	○	
Loss of pulses		○
Bruits over accessible arteries		○
Blood pressure discrepancy		○
Caudication of extremities		○
Ischaemic cardiac pain		○
Cardiomyopathy		○
Congestive cardiac failure		○
Valvular heart disease		○
Pericarditis		○
**7. Abdominal**	○	
Abdominal pain		○
Peritonitis		○
Blood in stools or bloody diarrhoea		○
Bowel ischaemia		○
**8. Renal**	○	
Hypertension >95th centile (for height)		○
Proteinuria >0.3 g/24h,>20mmol/mg creatinin		○
Haematuria ≥2+ or 5 rbc/hpf or red cell casts		○
GFR 50-80ml/min/1.73 m^2		○
GFR 15-49 ml/min/1.73 m^2		○
GFR <15 ml/min/1.73m^2		○
Rise in creatinine > 10% or Creatinine clearance (GFR) fall > 25%		○
**9. Nervous system**	○	
Headache		○
Meningitis/encephalitis		○
Organic confusion/cognitive dysfunction		○
Seizures (not hypertensive)		○
Stroke		○
Cord lesion		○
Cranial nerve palsy		○
Sensory peripheral neuropathy		○
Motor mononeuritis multiplex		○
**10. OTHER**	○	
		○
		○

NO NEW/WORSE DISEASE :

Tick here if there is no new/worse abnormality present in ANY of the systems above and active items represent low grade grumbling disease

Modified from Luqmani R.A., Bacon P.A., Moots R.J. et al (1994) BVAS in systemic necrotizing vasculitis. Quart J Med 87: 671-8
